# Increasing trend in the rate of infectious disease hospitalisations among Alaska Native people

**DOI:** 10.3402/ijch.v72i0.20994

**Published:** 2013-08-05

**Authors:** Robert C. Holman, Thomas W. Hennessy, Dana L. Haberling, Laura S. Callinan, Rosalyn J. Singleton, John T. Redd, Claudia A. Steiner, Michael G. Bruce

**Affiliations:** 1 Division of High-Consequence Pathogens and Pathology, National Center for Emerging and Zoonotic Infectious Diseases (NCEZID), Centers for Disease Control and Prevention (CDC), U.S. Department of Health and Human Services (USDHHS), Atlanta, GA, USA; 2 Arctic Investigations Program, NCEZID, CDC, USDHHS, Anchorage, AK, USA; 3 Alaska Native Tribal Health Consortium, Anchorage AK, USA; 4 Indian Health Service, USDHHS, Santa Fe, NM, USA; 5 Healthcare Cost and Utilization Project, Center for Delivery, Organization and Markets, Agency for Healthcare Research and Quality, Rockville, MD, USA

**Keywords:** Alaska Native, infectious disease, hospitalisations, Alaska, lower respiratory tract infection

## Abstract

**Objectives:**

To examine the epidemiology of infectious disease (ID) hospitalisations among Alaska Native (AN) people.

**Methods:**

Hospitalisations with a first-listed ID diagnosis for American Indians and ANs residing in Alaska during 2001–2009 were selected from the Indian Health Service direct and contract health service inpatient data. ID hospitalisations to describe the general US population were selected from the Nationwide Inpatient Sample. Annual and average annual (2007–2009) hospitalization rates were calculated.

**Results:**

During 2007–2009, IDs accounted for 20% of hospitalisations among AN people. The 2007–2009 average annual age-adjusted ID hospitalisation rate (2126/100,000 persons) was higher than that for the general US population (1679/100,000; 95% CI 1639–1720). The ID hospitalisation rate for AN people increased from 2001 to 2009 (17%, p<0.001). Although the rate during 2001–2009 declined for AN infants (<1 year of age; p=0.03), they had the highest 2007–2009 average annual rate (15106/100,000), which was 3 times the rate for general US infants (5215/100,000; 95% CI 4783–5647). The annual rates for the age groups 1–4, 5–19, 40–49, 50–59 and 70–79 years increased (p<0.05). The highest 2007–2009 age-adjusted average annual ID hospitalisation rates were in the Yukon-Kuskokwim (YK) (3492/100,000) and Kotzebue (3433/100,000) regions; infant rates were 30422/100,000 and 26698/100,000 in these regions, respectively. During 2007–2009, lower respiratory tract infections accounted for 39% of all ID hospitalisations and approximately 50% of ID hospitalisations in YK, Kotzebue and Norton Sound, and 74% of infant ID hospitalisations.

**Conclusions:**

The ID hospitalisation rate increased for AN people overall. The rate for AN people remained higher than that for the general US population, particularly in infants and in the YK and Kotzebue regions. Prevention measures to reduce ID morbidity among AN people should be increased in high-risk regions and for diseases with high hospitalisation rates.

Infectious diseases (IDs) have caused excessive morbidity and mortality among the American Indian and Alaska Native (AI/AN) population in the United States (1–4). Both hospitalisations and outpatient visits due to IDs have been disproportionately higher in the AI/AN population using the Indian Health Service (IHS) healthcare system when compared to the general US population (2, 5–8). These disparities affected AI/AN people of all ages, especially infants (<1 year of age) and older adults (3, 4, 7–9) . Studies of specific IDs, including lower respiratory tract infections (LRTIs), diarrhoea-related infections, and skin and ear infections, have shown a disparity between AI/ANs and the general US population (5–7, 10–13).

A high rate of ID hospitalisations has been reported in the AI/AN population in Alaska (2–4, 6–8, 10), and the overall infant ID hospitalisation rate in the IHS Alaska region has been reported higher than that for all other IHS regions and the general US population (2, 3). Respiratory disease has been implicated as a major health problem in the AN population, especially among young children (10, 12–17) . The age-adjusted LRTI hospitalisation rate for AN people was higher than that for the general US population during 1998–2006 (2). Furthermore, a greater disparity was seen among young children living in the Yukon-Kuskokwim (YK) region, where the rate of respiratory disease was much higher than that in other regions of Alaska (13). The lack of in-home water service in Alaska has been identified as one potential cause for the higher respiratory and skin infection rates (14–16).

The present study examines the epidemiology of ID hospitalisations among AN people using IHS/tribal health care system in Alaska, unlike most earlier studies that consider the AI/AN population within the IHS/tribal health care system in the United States. Rates of ID and specific ID group hospitalisations for AN people are analysed overall and for the regions in Alaska, along with rates for the general US population for comparison.

## Methods

This study was conducted using hospital discharge records for AI/ANs living in Alaska. Approximately 140,000 AN people are descendants of the indigenous population and represent about 20% of the Alaska population (18). The IHS federal healthcare services in Alaska are administered by regional AN-managed tribal health organisations with some statewide facilities and services shared and co-administered, such as the referral medical centre in Anchorage (19). Hospital discharge records for AN people were accessed from the IHS direct and contract health service inpatient dataset, IHS National Patient Information Reporting System (NPIRS), for 2001–2009. The IHS inpatient data consist of all hospital discharge records reported directly from IHS- or tribally-operated hospitals and community hospitals, which are contracted with IHS or tribes to provide healthcare services to eligible AI/AN persons (1).

Hospitalisation records for the overall general US population (all race groups) from the Nationwide Inpatient Sample (NIS) for 2001–2009 were analysed for comparison with the IHS/tribal AN population of the same time period (20). The NIS is a nationally representative survey of US short-term non-federal community hospitals conducted by the Healthcare Cost and Utilization Project in collaboration with participating states; the data and the weighting methodology for the NIS are described previously (20, 21). Hospital stays for births were excluded. The unit of analysis for the study was a hospitalisation.

The ID hospitalisations were defined by using a previously described ID classification method (2, 21), based on the *International Classification of Diseases, 9th revision, Clinical Modification* (ICD-9-CM) codes (22). First-listed ID hospitalisations were categorised into ID groups as follows: tuberculosis (010–018, 137); meningitis (027.0, 036, 320.0–321.3, 321.8); septicaemia (038, 449, 771.81, 995.90–995.94); HIV/AIDS (042–044, 279.1); hepatobiliary disease (070, 095.3, 573.1, 573.2, 576.1); mycoses (110–118); infections of the heart (093, 391, 392.0, 393, 394.1, 395.0–395.2, 397.1, 397.9, 398, 421, 422.0, 424.9); upper respiratory tract infections (032.0–032.3, 034.0, 098.6, 101, 460–465, 473.0–474.0, 475); LRTIs (022.1, 031.0, 033, 095.1, 466, 480–488, 510, 511.1, 513, 517.1, 770.0); abdominal and rectal infections (095.2, 098.7, 540–542, 566, 567.0–567.2, 567.38, 569.5); kidney, urinary tract and bladder infections (KUTBs) (095.4, 099.4, 590, 595.0, 597, 598.0, 599.0); skin and soft tissue infections (SSTIs) (680–686); enteric infections (001–009, 022.2); viral central nervous system infections (045–049, 059.00, 059.09–059.12, 059.19); infection due to internal prosthetic device, implant and graft (996.6, 999.31); post-operative infection (780.62, 998.5); osteomyelitis, periostitis and other infections involving bone (730); inflammatory disease of female pelvic organs (614.0–614.5, 616.0–616.1, 616.3–616.4, 625.71); and infections related to pregnancy, childbirth, or puerperium (634.0, 635.0, 636.0, 637.0, 638.0, 639.0, 646.5, 646.6, 647, 655.3, 658.4, 659.3, 670, 675) (2, 21). First-listed ID hospital discharge records were selected and examined overall, by age, sex, Alaska IHS service area (region) and specific ID group. Alaska regions were defined as follows: Anchorage, Barrow, YK, Bristol Bay, Kotzebue, Mt. Edgecumbe, Interior Alaska, Annette Island and Norton Sound (23). All regions are included in the overall analysis, while not all analyses are provided for each region due to non-reporting and small numbers.

Annual and average-annual hospitalisation rates were calculated as the number of hospitalisations per 100,000 corresponding population. Rates were calculated for first-listed ID hospitalisations and ID groups. Analyses were restricted to records in which the first-listed diagnosis was an ID diagnosis, unless otherwise specified. To analyse the most recent time period, average annual ID hospitalisation rates were calculated and analyses were presented for 2007–2009. In addition, rate trends were analysed for 2001–2009. The IHS Alaska user population for the fiscal years 2001–2009 was used as the denominator for corresponding populations (1). The user population includes all people who received IHS-funded healthcare service at least once during the previous 3 years (1, 24). Age-adjusted hospitalisation rates for both the IHS Alaska population and the general US population were calculated using the direct method, with the 2000 projected US population as the standard, (25) to adjust for differences in the age distribution of populations. Trends in the annual hospitalisation rate during 2001–2009 were assessed using Poisson regression (26). Month of hospital admission and length of stay (LOS) were examined overall and by region. Median and interquartile range (IQR) for age and LOS are presented. Comparisons of age distribution between groups were made using a Wilcoxon rank-sum test (27). Categorical data were analysed using the Chi-square test. Statistical tests were conducted using p<0.05 as the significance level.

For the general US population, hospitalisation rates with 95% confidence intervals (CIs) were calculated as the weighted number of hospitalisations with population denominators determined from the National Center for Health Statistics bridged-race population estimates (20, 28). All calculations take into account the sampling design of the NIS; hospitalisation and proportional estimates and corresponding standard errors (SEs) were calculated using SUDAAN software (29). To test for a linear trend in the annual hospitalisation rate for the general US population during 2001–2009, a weighted least squares technique that accounts for the NIS sample design was used (30).

## Results

### Trends in infectious disease hospitalisations, 2001–2009

**Fig. 1:**
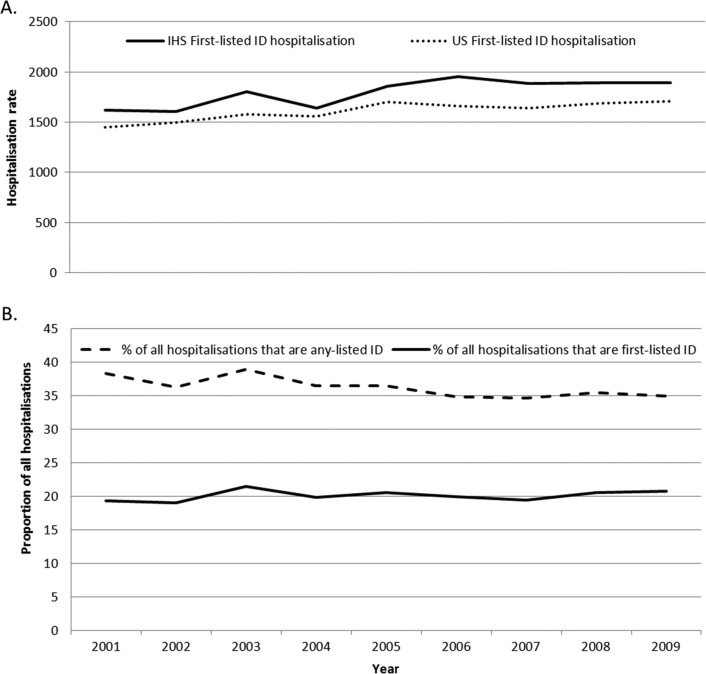
A) Annual rates of first-listed infectious disease (ID) hospitalisations among Alaska Native people, Alaska, and for the general US population, 2001–2009 and B) Annual proportions of first-listed and of any-listed ID hospitalisations in all hospitalisations among Alaska Native people, Alaska, 2001–2009. *First-listed ID hospitalisations are hospitalisations where the first diagnosis is an ID, any-listed ID hospitalisations are hospitalisations with an ID ICD-9-CM code listed anywhere on the discharge record. Rate per 100,000 persons of corresponding group. Rates for Alaska Native people were determined from the Indian Health Service (IHS) direct and contract health service inpatient dataset. Rates for the general US population used the Nationwide Inpatient Sample (NIS) to represent all of the US population.

The annual ID hospitalisation rate for AN people increased during 2001–2009 (17% from 2001 to 2009; p<0.001); the rate for the general US population similarly increased (18%, 95% CI 12–25%; p<0.001) (Fig. 1A). The ID hospitalisation rate for both AN males (14%, p=0.004) and AN females (20%, p=0.002) increased. Infants were the only age group to have a declining rate (15%, p=0.03); the age groups 1–4 (22%), 5–19 (31%), 40–49 (30%), 50–59 (16%) and 70–79 (27%) years each had a significant increase (p<0.05). The trend for the general US infant population also decreased (22%, 95% CI 6–37%, p<0.01). A significant rate increase was seen in the following regions, Kotzebue (67%; p<0.001), Mt. Edgecumbe (42%; p=0.01) and Norton Sound (169%; p=0.003). The rate for YK remained high and did not significantly change over time. For the ID groups among AN people, significant rate increases were seen for LRTI (16%; p=0.04) and septicaemia (172%; p<0.001); a rate change was not seen in the other ID groups with sufficient numbers. For the general US population, the rate for LRTI decreased (7%, 95% CI 1–12%, p<0.01) and the septicaemia rate increased (134%, 95% CI 117–152%, p<0.001).

### Infectious diseases hospitalisations, 2007–2009

Infectious diseases accounted for 20% of all hospitalisations among AN people during 2007–2009; the proportion for the general US population was 14.5% (95% CI 14.3–14.7%). The annual proportion of ID hospitalisations among all hospitalisations was relatively stable throughout 2001–2009 (Fig. 1B); the proportion among all general US hospitalisations was also stable. The proportion of all ID hospitalisations ranged from 11% to 25% among Alaska regions. By age group for AN people, the proportion of ID hospitalisations among all hospitalisations for 2007–2009 was highest among young children (51% in infants and 57% in 1–4 year olds).

For 2007–2009, the median age for ID hospitalisations for AN people was 28 years (IQR 2, 54 years). Thirty-nine percent of the ID hospitalisations occurred in those <20 years of age. The highest regional median age was in Mt. Edgecumbe (46 years; IQR 22, 66) and lowest median age was in YK (13 years; IQR 0, 47). The overall and regional median ages for AN people were younger than the median age of ID hospitalisations for the general US population (57.9 years, 95% CI 57.1–59.4; IQR 34.2, 76.4).

### Infectious disease hospitalisation rates, 2007–2009

The 2007–2009 average annual age-adjusted ID hospitalisation rate (2125.7/100,000) was higher for AN people than for the general US population (1628/100,000, 95% CI 1610–1646; Table I). The overall age-adjusted rates for AN males and AN females were similar. The ID hospitalisation rate was highest for AN infants, followed by AN people ≥80 years of age. The rate for AN infants (15106.2/100,000) was much higher than the rate for the general US infant population (5215/100,000, 95% CI 4783–5647; Table I). Both AN males and females had a higher age-adjusted rate than those in the general US population. The regional age-adjusted ID hospitalisation rate was highest for YK, followed by Kotzebue and Barrow (Table I). The AN infant rate was highest in YK and Kotzebue. In all regions, the overall age-adjusted ID hospitalisation rates were greater than the rate for the general US population. The age-specific ID hospitalisation rates for AN males and AN females were similar for each age group, except for AN infants where the rate for males was higher than for females (16568 and 13528/100,000, respectively) and for the 20–29 year age group where the rate for females was higher than the rate for males (1342 and 906/100,000, respectively).

**Table I:** Infectious disease hospitalisation rates among Alaska Native people by sex, age group and select regions, Alaska (IHS), and for the general US population (NIS), 2007–2009^a^

Characteristic	Anchorage	Barrow	Yukon-Kuskokwim	Bristol Bay	Kotzebue	Mt. Edgecumbe	Norton Sound	Alaska Native people overall	US overall rate (95% CI)
Age-adjusted rates									
Total	1691.0	2712.8	3491.5	1806.9	3433.1	2189.1	2298.7	2125.7	1628.0 (1609.6–1646.3)
Sex									
Male	1635.0	2758.6	3636.6	1915.1	2649.1	2239.9	2106.0	2109.2	1643.3 (1623.6–1662.9)
Female	1733.7	2710.6	3360.2	1662.2	4221.4	2158.8	2497.5	2139.9	1616.6 (1598.8–1634.4)
Age group (years): age-specific rates									
<1	5574.7	17061.6	30422.0	11878.5	26697.5	10173.2	20057.7	15106.2	5215.0 (4783.4–5646.6)
1–4	1187.5	3554.0	5451.5	1175.6	3991.6	1642.5	3362.0	2480.5	1247.9 (1150.3–1345.5)
5–19	561.6	1085.2	1251.8	627.2	1104.7	653.3	782.7	726.9	523.7 (491.9–555.5)
20–29	867.1	1611.4	2071.2	761.7	1734.8	1566.8	1039.8	1127.2	779.1 (754.0–804.2)
30–39	995.4	945.0	2252.9	782.4	1925.0	1304.7	1703.9	1216.3	909.8 (879.5–940.0)
40–49	1826.3	1940.0	2303.8	1478.1	2402.0	1558.9	1651.0	1727.2	1161.4 (1125.8–1197.0)
50–59	2027.2	2938.8	2180.4	1536.6	2923.6	1921.9	2667.9	1936.2	1618.6 (1574.7–1662.5)
60–69	2884.5	3260.9	4415.5	3082.2	3121.4	3953.8	3132.7	3035.7	2529.1 (2467.3–2590.9)
70–79	3958.2	6925.2	9316.2	6060.6	10895.5	6551.2	4918.0	5525.6	4720.0 (4604.3–4835.8)
≥80	6336.9	12562.8	12488.4	7279.7	15596.3	8529.7	6045.3	7838.7	9348.0 (9093.6–9602.4)

a Rate per 100,000 persons of corresponding group. Rates for Alaska Native people were determined from the Indian Health Service (IHS) direct and contract health service inpatient dataset. Rates for the general US population used the Nationwide Inpatient Sample (NIS) to represent all of the US population. The 2007–2009 average annual regional populations for the Alaska Native people were as follows: Anchorage (57082), Barrow (4358), Yukon-Kuskokwim (24707), Bristol Bay (5277), Kotzebue (7038), Mt. Edgecumbe (15073), Norton Sound (8281), Interior Alaska (12959) and Annette Island (1415). Specific Interior Alaska and Annette Island regions are included in the overall analysis, but not presented individually due to non-reporting and small numbers.

### Hospital month of admission and length of stay, 2007–2009

The proportion of ID admissions by month for AN people did not vary substantially overall (7–9%) or within each region. The median LOS for ID hospitalisations was 3 days (IQR 2, 6 days) and was similar for males and females. The median LOS ranged from 3 to 4 days for most regions.

Overall, 16% of hospital admissions were for <2 days. This proportion varied by region, Anchorage (14%), Barrow (20%), YK (13%), Bristol Bay (17%), Kotzebue (20%), Mt. Edgecumbe (24%) and Norton Sound (17%); the Anchorage and YK regions had a higher proportion of hospitalisations ≥2 days than each of the other regions (p<0.05). The proportion for the general US population was 13% (SE=0.1%).

### Infectious disease groups and diagnoses, 2007–2009

LRTI was the primary diagnosis on 39% of the ID hospitalisations among AN people. The proportion of ID hospitalisations that were due to LRTI was high in Norton Sound (56%), YK (47%), Kotzebue (47%) and Barrow (44%) compared to the general US population (27.5%, 95% CI 27.1–27.9%). The proportion for the Anchorage area (27%) was similar to the general US population. The LRTI hospitalisations among AN people were primarily (61%) listed as “pneumonia, organism unspecified.”

The highest average annual hospitalisation rate among all ID groups among AN people was for LRTI (Table II). The overall average annual age-adjusted LRTI rate for AN people was almost twice that of the general US population; all Alaska regions with the exception of two exceeded the general US population rate. The regional age-adjusted LRTI hospitalisation rates were highest for AN people in YK, Kotzebue and Norton Sound (Table III). The LRTI rate was highest for AN infants (11190/100,000) compared to other AN age groups and to the general US infant population rate (3018/100,000; 95% CI 2779–3258). The infant LRTI rate was highest in YK (22718/100,000) followed by Kotzebue (19444/100,000) and Norton Sound (17172/100,000).

**Table II:** Age-adjusted hospitalisation rates for select infectious disease groups among Alaska Native people in Alaska and for the overall general US population, 2007–2009^a^

	Alaska Native people	United States
	
Infectious disease group	Age-adjusted rate	Age-adjusted rate (95% CI)
Lower respiratory tract infection	812.9	445.3 (439.4–451.2)
Skin and soft tissue infections	354.0	198.3 (195.7–200.8)
Abdominal and rectal infection	148.7	117.2 (114.9–119.5)
Kidney, urinary tract and bladder infection	190.2	174.5 (172.0–177.0)
Septicaemia	163.1	232.0 (228.2–235.8)
Upper respiratory tract infection	58.3	33.1 (32.2–34.0)
Infection related to pregnancy, childbirth, or puerperium^b^	117.3	71.6 (69.5–73.8)
Postoperative infection	51.1	50.3 (49.5–51.2)
Enteric infection	40.1	74.9 (73.6–76.2)
Infection due to internal prosthetic device, implant, or graft	32.1	70.7 (69.5–71.9)
Osteomyelitis, periostitis and other infections involving bone	20.7	18.4 (18.0–18.8)
Infections of the heart	22.5	14.3 (13.9–14.8)
Viral central nervous system infection	10.8	11.0 (10.6–11.3)

a Rate per 100,000 persons of corresponding group. Infectious disease groups with at least 50 hospitalisations were displayed. Rates for Alaska Native people were determined from the Indian Health Service (IHS) direct and contract health service inpatient dataset. Rates for the general US population used the Nationwide Inpatient Sample (NIS) to represent all of the US population.

b Female only.

**Table III:** Hospitalisation rates for select infectious disease groups among Alaska Native people by region, Alaska, 2007–2009^a^

	Anchorage		Barrow		Yukon-Kuskokwim		Bristol Bay	
				
Infectious disease group	No.	Rate	No.	Rate	No.	Rate	No.	Rate
Lower respiratory tract infection	637	472.6	154	1094.3	1197	1513.0	107	744.8
Skin and soft tissue infections	389	266.5	53	461.8	520	806.7	39	302.2
Abdominal and rectal infection	240	125.5	33	217.1	182	227.9	27	149.6
Kidney, urinary tract and bladder infection	192	186.7	22	225.2	146	283.9	19	153.3
Septicaemia	239	222.8	18	166.0	76	137.9	14	113.1
Upper respiratory tract infection	52	25.1	15	77.6	69	65.3	*	*
Infection related to pregnancy, childbirth, or puerperium^b^	132	116.2	14	223.6	64	162.8	*	*
	Kotzebue		Mt. Edgecumbe		Norton Sound		Overall Alaska	
	
Infectious disease group	No.	Rate	No.	Rate	No.	Rate	No.	Rate

Lower respiratory tract infection	330	1460.7	277	761.2	321	1245.4	3029	812.9
Skin and soft tissue infections	74	433.0	127	309.6	58	266.9	1268	354.0
Abdominal and rectal infection	45	213.9	73	168.4	40	144.8	645	148.7
Kidney, urinary tract and bladder infection	61	442.6	80	200.0	21	86.1	546	190.2
Septicaemia	20	124.6	57	158.8	20	92.2	465	163.1
Upper respiratory tract infection	33	116.2	83	172.0	25	73.3	286	58.3
Infection related to pregnancy, childbirth, or puerperium^b^	32	301.6	16	55.2	20	67.8	285	117.2

a Rate per 100,000 persons of corresponding group. Rates for Alaska Native people were determined from the Indian Health Service (IHS) direct and contract health service inpatient dataset. The Interior Alaska and Annette Island regions are included in the overall analysis, but not presented due to non-reporting and small number of hospitalisations.

b Female only.

* Less than 10 hospitalisations.

The second most common ID group among AN people was SSTIs (Tables II and III), with a higher proportion of the ID hospitalisations (16%) than that for the general US population (12.1%; 95% CI 11.9–12.3%). The proportion of ID hospitalisations that were due to SSTI ranged from 5% to 20%, by region. The age-adjusted rate for males was higher than that for females in almost all regions. The regional age-adjusted SSTI rate for AN people was highest in YK, followed by Barrow and Kotzebue regions (Table III). Kidney urinary tract and bladder infections accounted for 7% of the ID hospitalisations and accounted for a lower proportion of IDs than for the general US population (10.8%; 95% CI 10.6–11%). The KUTB infection rate for females was higher than that for males. The age-adjusted KUTB rate was highest in Kotzebue followed by YK (Table III).

## Discussion

In the present evaluation of ID hospitalisation records, the rate increased during 2001–2009 for both the AN people and the general US population. This increase among AN people was seen among both males and females. The ID hospitalisation rate increased during 2001–2009 in some age groups, but decreased for AN infants even though AN infants had the highest rate among all age groups. The rate also decreased for the general US infant population. Four of the Alaska regions showed an ID hospitalisation rate increase. The rate for YK region, which had the highest ID hospitalisation rate, remained unchanged during the study period. The 2007–2009 age-adjusted ID hospitalisation rate for AN people was higher than that for the general US population. Regionally, the ID hospitalisation rate was highest for the YK region, followed by the Kotzebue and Barrow regions.

The on-going ID disparities among AN people, especially in rural regions and among infants, may be attributed to household crowding, household air pollution, lack of in-home water service and consequences of poverty (14–16, 31, 32). Despite considerable progress, approximately 18% of rural AN homes still lack in-home water and sanitation services (personal communication, Matt Dixon, AN Tribal Health Consortium, January 19, 2012). Lack of access to in-home water service, which leads to water rationing and reduced access to water for hand and body hygiene, has been associated with increased respiratory disease rates among AN children (14–16, 31).

Among the age groups, AN infants showed the greatest ID disparity compared to the general US infant population, with rates nearly 3-fold higher. Encouragingly, the hospitalisation rate decreased by 15% during 2001–2009 for AN infants. However, AN infants have the highest ID hospitalisation rate of all age groups. The most common cause of AN infant hospitalisations is LRTIs, and rates in Alaska have been higher than rates for AI/ANs from other IHS regions (7, 10, 13).

The age-adjusted average annual LRTI hospitalisation rate for AN people was the highest among ID groups and was almost 2-fold higher than the rate for the general US population. Earlier studies of AI/ANs have found higher hospitalisation rates for LRTI and bronchiolitis in Alaska compared to the general US population, (10, 12, 13) including an increase in pneumonia hospitalisations among older AN adults (9). The LRTI hospitalisation rates varied considerably among Alaska regions; reasons for this are not clear. However, LRTI was still the most common cause of hospitalisation for each region. High rates of respiratory infections have been previously reported among children for the YK region (13, 14, 33).

The present study has some limitations. Hospital diagnoses may be incomplete or inaccurate due to use of ICD-9-CM coding to identify ID hospitalisations, and diagnostic coding may vary by practice. The first-listed diagnosis was used to identify ID hospitalisations in an attempt to limit misclassification of non-ID hospitalisations. However, this conservative method means that any true ID hospitalisations in which an ID was not the first-listed diagnosis were not included. The unit of analysis was a hospitalisation, therefore any multiple hospitalisations and/or hospital transfers for a patient's event were included. Certain ID groups may be better expressed by using a different denominator for their rate calculation such as analysing infection related to pregnancy, childbirth or puerperium with pregnancies as the denominator or post-operative infections with number of procedures as the denominator; however, this was not possible in the present study as data was not available.

The denominator for the AN population in the present study is based on the number of AI/ANs who utilise the tribal healthcare system in Alaska and may not include all Alaska Native people who are eligible for healthcare at tribal facilities which would underestimate the denominator. However, some additional limitations that we are aware of indicate that our reported rates of hospitalisations for AN people may be an underestimate. AN patients who are eligible for healthcare within the tribal healthcare system may receive care outside the system; therefore, their hospitalisations would not be included in the present study. Also, reporting of hospitalisations for Alaska regions to IHS National Patient Information Reporting System, as well as reporting for the NIS, may not be complete. In an earlier investigation, 8 influenza hospitalisations were found in the Interior Alaska region in September and October of 2009 (34), while there were none reported to NPIRS for that period. The reasons for and the extent of the under-reporting are not known and deserve further investigation. The denominator used to estimate rates for AN people in the present study was estimated as the number of AN people who used the IHS/tribal healthcare system, whereas the US census data were used as the denominator to estimate the rates for the general US population, which may have affected the comparability of the rates for the two populations. However, the difference between the IHS user population and the corresponding census data has been reported to be small (24). Further study of IDs using Alaska hospital discharge data, along with IHS/tribal inpatient data, would allow for additional examination of disparities and other characteristics of ID hospitalisation within Alaska.

## Conclusion

The rate of ID hospitalisations increased for AN people during 2001–2009 and ID hospitalisation rates in all Alaska regions remain higher than the general US population. The rate disparity is substantially greater in the YK and Kotzebue regions, although in-home water and sanitation service progress is being made in rural Alaska. The disparity in hospitalisation rates is greatest for AN infants, primarily due to LRTIs. It is important to increase efforts and to implement strategies to prevent or reduce the ID health disparities through public health interventions and education to improve the health status of AN people, as well as continued improvements in areas such as access to in-home water. The strategies should focus on age groups and regions at high risk of ID hospitalisation, along with illnesses contributing most to the substantial health disparity.
